# Targeted Temperature Management for In-hospital Cardiac Arrest Caused by Thyroid Storm: A Case Report

**DOI:** 10.3389/fcvm.2021.634987

**Published:** 2021-07-21

**Authors:** Yuanwei Fu, Hongxia Ge, Yumei Zhang, Yan Li, Bingyao Mu, Wen Shang, Shu Li, Qingbian Ma

**Affiliations:** Department of Emergency Medicine, Peking University Third Hospital, Beijing, China

**Keywords:** thyroid storm, ventricular arrhythmia, cardiac arrest, cardiopulmonary resuscitation, targeted temperature management

## Abstract

**Background:** Malignant ventricular arrhythmias caused by thyroid storm, such as ventricular tachycardia (VT) or ventricular fibrillation (VF), which are life-threatening, are rare. We report the case of a patient who suffered from cardiac arrest caused by thyroid storm and the rare VF; the patient showed a favorable neurologic outcome after receiving targeted temperature management (TTM) treatment by intravascular cooling measures.

**Case presentation:** A 24-year-old woman who had lost 20 kg in the preceding 2 months presented to the emergency department with diarrhea, vomiting, fever, and tachycardia. Thyroid function testing showed increased free triiodothyronine (FT3) and free thyroxine (FT4), decreased thyroid-stimulating hormone (TSH), and positive TSH-receptor antibody (TRAB). She was diagnosed with hyperthyroidism and had experienced sudden cardiac arrest (SCA) due to ventricular fibrillation (VF) caused by thyroid storm. The patient was performed with targeted temperature management (TTM) by intravascular cooling measures. Regular follow-up in the endocrinology department showed a good outcome.

**Conclusions:** Our case not only suggests a new method of cooling treatment for thyroid storm, but also provides evidence for the success of TTM on patients resuscitated from in-hospital cardiac arrest (IHCA) who remain comatose after return of spontaneous circulation (ROSC).

## Introduction

Thyroid storm is an endocrine emergency that is characterized by multiple organ failure due to severe thyrotoxicosis. The most common arrhythmias caused by thyrotoxicosis are sinus tachycardia and atrial fibrillation (AF), which present in 28% of patients. Malignant ventricular arrhythmias, such as ventricular tachycardia (VT) or ventricular fibrillation (VF), which are life-threatening, are rare (1). We thus report a case of cardiac arrest induced by torsade de pointes (TdP) and VF due to thyroid storm, in which the patient was treated with intravascular cooling measures to protect neurological function. We investigate the mechanism of malignant ventricular arrhythmia caused by thyrotoxicosis and explore hypothermia therapy for patients who suffer from cardiac arrest caused by thyroid storm.

## Case Presentation

A 24-year-old Asian woman presented to the emergency department, who had no previous medical history, psychosocial history and family history. She had lost 20 kg in the previous 2 months with no other symptoms, but 10 days before admission, the patient presented to emergency department because of diarrhea for unknown reasons, accompanied by vomiting and fever. Then physical examination revealed body temperature at 37.4°C, heart rate 160 bpm, and blood pressure 128/108 mmHg. The patient was successively treated with latamoxef, moxifloxacin for anti-infection treatment, and metoprolol for heart rate control. She had to present to emergency department again when her condition did not improve after 4 days of continuous treatment and her heart rate continued to fluctuate between 100 and 160 bpm. Then physical examination showed no other positive findings except grade II thyroid enlargement. Thyroid function testing showed increased free triiodothyronine (FT3) and free thyroxine (FT4), decreased thyroid-stimulating hormone (TSH), and positive TSH-receptor antibody (TRAB). Thyroid ultrasound revealed diffuse thyroid parenchyma lesions. Based on these findings, the patient was diagnosed with hyperthyroidism, and received treatment with metoprolol 25 mg b.i.d. as well as methimazole 30 mg q.d. orally for 4 days in emergency observation room.

No significant remission in her symptoms of vomiting and diarrhea during or following treatment had been observed. In the emergency observation room, the patient lost consciousness and the electrocardiogram (ECG) monitor showed VF; she was thus immediately transferred to the resuscitation room for cardiopulmonary resuscitation (CPR). After 16 min of CPR, return of spontaneous circulation (ROSC) was observed, with Glasgow coma scale (GCS) <8. Physical examination showed a heart rate of 166 bpm, blood pressure of 98/73 mmHg, and body temperature of 39.1°C. The patient was diagnosed with thyroid storm based on clinical presentation and her Burch-Wartofsky score of 50 when first presented to the emergency department. She was admitted to the emergency intensive care unit (EICU) for therapeutic hypothermia treatment.

Laboratory data, including complete blood cell counts, liver function, renal function, cardiac enzyme, and electrolyte levels were all within normal limits when the patient first presented to the emergency department. Four days after emergency treatment, a thyroid function test showed the following levels: FT3 > 20.00 (2.3–4.2) pg/ml, FT4 > 12.00 (0.89–1.80) ng/dl, TSH < 0.008 (0.55–4.78) uIU/ml; TRAb > 40 (<60) U/L; thyroglobulin antibodies (TGAb) 222.0 (<60) U/ml, and thyroid microsomal antibody (TMAb) 34.3 (<60) U/ml. Thyroid ultrasound revealed diffuse thyroid parenchyma lesions. Based on these findings, the patient presented with thyroid storm due to uncontrolled Graves' disease.

**Figure 1:**
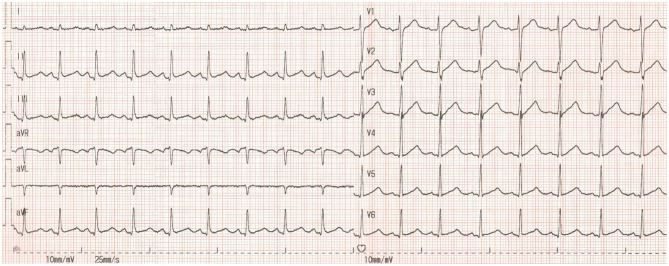
The patient's ECG before cardiac arrest. HR = 105 bpm, RR = 0.568 s, QT = 0.375 s. QTc = 0.498 s, QTc = QT /(RR)1/2.

**Figure 2:**
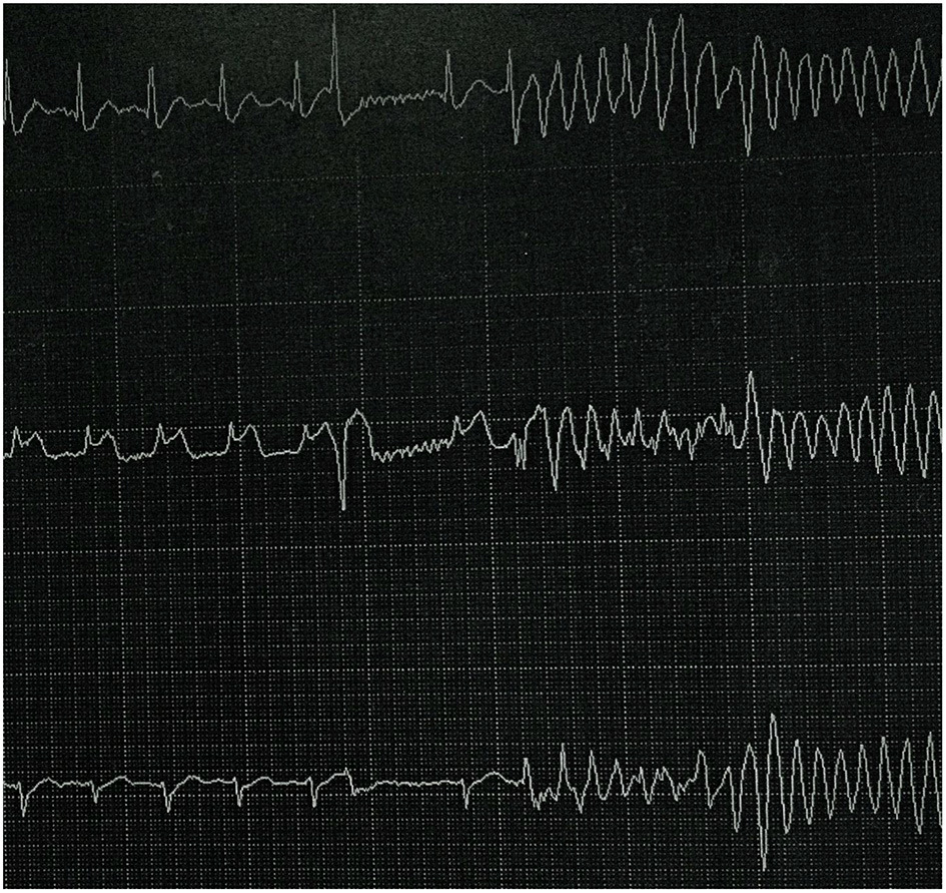
ECG monitor data after cardiac arrest. ST-segment elevation induced TdP and VF after R-on-T phenomenon.

The patient's ECG before cardiac arrest revealed sinus tachycardia and a prolonged QTc. The measured QTc interval was 0.498 s at a heart rate of 105 bpm (Figure 1). We reviewed ECG monitor data after cardiac arrest (Figure 2) and found that transient ST-segment elevation induced TdP and VF after the R-on-T phenomenon, which suggested that the cause of cardiac arrest in the patient was malignant ventricular arrhythmia. The patient's echocardiogram before cardiac arrest showed left ventricular ejection fraction (LVEF) was 74% and decreased to 40% after ROSC.

On admission to EICU for thyroid storm, the patient was initiated on propylthiouracil 400 mg which could inhibit both thyroid hormone synthesis and peripheral conversion of T4–T3, and maintained on 200 mg t.i.d. The dose was then gradually reduced to 100 mg t.i.d. The patient was also treated with propranolol 40 mg t.i.d. to achieve adequate control of heart rate, which could also block the stimulating effects of thyroid hormones on the heart and inhibit the transformation of peripheral T4–T3. The patient was also started on hydrocortisone 100 mg q.8h. for 3 days, followed by 50 mg q.8h. for 2 days and then 50 mg q.12h. for 5 days, which could prevent adrenocortical dysfunction. The hydrocortisone was discontinued on day 10 after admission. The patient exhibited favorable outcome, and was discharged on a medication regimen including propylthiouracil 100 mg t.i.d. and propranolol 20 mg t.i.d.

**Figure 3:**
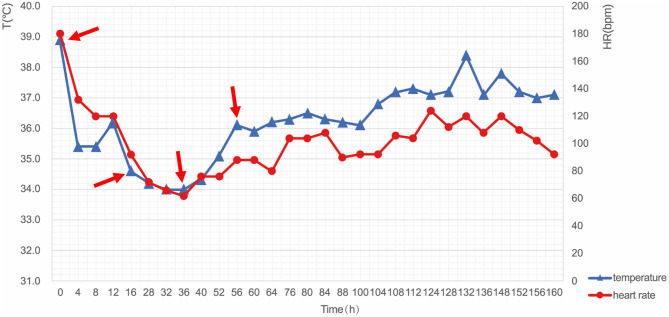
Changes in body temperature and heart rate during TTM. The key moments included: start of cooling (Hour 0, 38.9°C), reaching the target temperature (Hour 12, 34.0°C), start of rewarming (Hour 36, 33.8°C), and end of rewarming (Hour 56, 36.5°C). T, temperature; HR, heart rate; TTM, targeted temperature management.

After resuscitation, the patient had shown coma and high fever with a body temperature of 39.1°C; consequently, mild hypothermia therapy was started within the first 4 h after resuscitation and targeted temperature management (TTM) was performed by intravascular cooling measures to protect neurological function. CoolGard 3000 system and CoolLine catheter were used to provide stable TTM. The target temperature was set to 34°C. The patient's temperature dropped to 35.4°C 4 h later, and fluctuated around the target temperature at hour 12. During the following 24 h, the patient was maintained in a hypothermic state at 34°C followed by controlled rewarming (0.1°C/h). After a 72-h maintenance period, endotracheal intubation and the intravascular cooling catheter were removed. The patient recovered consciousness but had intermittent fever, and thus was treated with an ice blanket as well as an ice cap until her temperature returned to normal (Figure 3).

The patient's condition improved gradually, whereupon she was transferred from EICU on day 10 after admission. Cardiac MRI was performed before discharge, which showed LVEF was 72% and right ventricular ejection fraction (RVEF) was 66%. Regular follow-up in the endocrinology department showed a good outcome.

## Discussion

Thyroid storm is a life-threatening condition that has a mortality of 10–20%, which thus requires rapid diagnosis and emergent treatment (2, 3). In our case, the patient had a definite diagnosis of hyperthyroidism when she initially presented to the emergency department. Indications that thyroid storm should be considered included her Burch-Wartofsky score of 50, fever (temperature 37.4°C), tachycardia (heart rate 160 bpm), moderate gastrointestinal-hepatic dysfunction (vomiting and diarrhea), and precipitating event (irregular antithyroid drugs). We reviewed her ECG monitor data after cardiac arrest and found TdP and VF, which suggested that the cause of cardiac arrest was malignant ventricular arrhythmia. Severe thyrotoxicosis has been shown to increase cardiac excitability and induce coronary vasospasm, which may lead to ventricular arrhythmias.

In general, the most common arrhythmia caused by thyrotoxicosis is supraventricular arrhythmia. Malignant ventricular arrhythmias, such as VT or VF, are rare, and usually occur in patients with underlying heart disease. The density of myocardial adrenergic binding sites has been shown to be enhanced by thyroid hormones. In addition, thyroid hormones induce a rate-dependent lengthening of the Purkinje fiber action potential while the ventricular action potential is shortened. These changes can consequently enhance dispersion of myocardial repolarization, facilitate reentry arrhythmia and induce VF. Thyrotoxicosis may also affect myocardial electrical stability: increased excitability related to triggered activity can result in premature ventricular beats that often induce malignant arrhythmias (1). In addition, severe hypokalemia, coronary vasospasm, and prolonged or shortened QT interval may also induce malignant ventricular arrhythmias.

Review of ECG monitor data showed transient ST-segment elevation, indicating that coronary vasospasm should be considered. The patient was a young woman who did not have any risk factors for coronary heart disease. The electrocardiogram showed transient ST-segment elevation without pathological Q wave and neither echocardiogram nor cardiac MRI showed signs of myocardial infarction. The patient's troponin level increased to 0.51 ng/ml after resuscitation, which was considered to be relevant to myocardial injury during cardiopulmonary resuscitation. Therefore, we believed that the ST segment elevation was secondary to coronary spasm and that's why a cardiac catheterization was not performed. Similar cases have been reported in past research (4). Coronary vasospasm associated with thyrotoxicosis must be considered in the patient without any cardiac risk factors. Coronary vasospasm in thyrotoxicosis might result from an amplified sensitivity to norepinephrine and/or a diminished response to nitric oxide-mediated vasodilatation in the coronary arteries (5). Hyperthyroidism enhances the sensitivity of the vascular contractile responses to catecholamines, which can induce coronary vasospasm.

On the other hand, the ECG before cardiac arrest showed a mild prolonged QTc interval, which may induce ventricular arrhythmias. In thyrotoxicosis, the activity of cardiac Na^+^/K^+^ ATPase is increased, resulting in increased intracellular potassium, membrane hyperpolarization, and prolonged repolarization, all of which cause QTc prolongation. However, this theory has not yet been confirmed (6, 7). The utilization of quinolones may also exacerbate the QTc prolongation.

The patient was diagnosed with thyroid storm complicated with coma and hyperpyrexia after resuscitation. Based on the necessity of cooling and protecting neurologic function, we adopted intravascular cooling measures for TTM. High fever often occurs in thyroid storm due to thermoregulatory dysfunction and conventional cooling measures are often ineffective. Fever can increase metabolic rate and adrenergic response, which thus exacerbate multiple organ failure. Since the control of fever may reduce adverse effects on the central nervous system and cardiovascular function, cooling measures must be needed for thyroid storm patients with high fever. The 2016 Japan Thyroid Association and the Japan Endocrine Society (JTA/JES) guidelines recommend aggressive cooling, including use of acetaminophen and surface cooling devices, for patients with thyroid storm, but do not mention invasive intravascular cooling (2).

The neuroprotective efects of TTM occur through the following mechanism (8): (1) reducing brain metabolism and lowering the intracranial pressure; (2) reducing the initiation of brain cell apoptosis and necrosis; (3) decreasing the local release of lactate and excitotoxic compounds; (4) reducing the inflammatory response of brain tissue and systemic inflammatory response; (5) reducing the production of oxygen free radicals; (6) decreasing cerebral capillary permeability. The 2020 American Heart Association Guidelines for Cardiopulmonary Resuscitation and Emergency Cardiovascular Care recommend all comatose adult patients with ROSC after cardiac arrest should start TTM as soon as possible, with a target temperature between 32 and 36°C, then maintain constantly for at least 24 h (9). Recent studies have confirmed that TTM can improve neurological outcome in survivors resuscitated from out-of-hospital cardiac arrest (OHCA) and who remain comatose after ROSC (8, 10). However, whether therapeutic hypothermia has neuroprotective effects in in-hospital cardiac arrest (IHCA) patients is controversial (11). In our case, the patient was treated with advanced intravascular cooling devices in time. Her cerebral performance category (CPC) was one when she was discharged from hospital with good neurologic outcome.

The new intravascular cooling device has favorable effect during the induction phase and maintenance phase of TTM. Compared with conventional cooling methods, the advanced methods with servo-regulated cooling device can provide more rapid, more stable, more accurate therapeutic hypothermia and better control during rewarming to optimize TTM. This is the first time that intravascular cooling technique has been applied to both thyroid storm and cardiac arrest in China.

2017 AHA/ACC/HRS Guideline recommend implantable cardioverter defibrillator (ICD) therapy in patients with ischemic heart disease, who either survive cardiac arrest due to VF or hemodynamically unstable sustained VT or stable sustained VT, after any reversible cause is excluded (12). Results from a multicenter observational study of patients who underwent ICD suggest that ICD therapy should be strongly recommended for patients with coronary spasm and fatal ventricular arrhythmia even if they had no obstructive coronary artery disease (13). However, the use of ICD in patients with fatal ventricular arrhythmias due to coronary spasm remains controversial because coronary spasm can be controlled by medication (14). Therefore, in the absence of structural heart disease, we consider ICD is not indicated for the patient with ventricular arrhythmias that are due to completely reversible primary disease, but rather that the hyperthyroidism should be controlled actively.

## Conclusion

We report a case of IHCA caused by thyroid storm. Advanced cooling techniques were performed immediately for TTM. Eventually, the patient was discharged from hospital with good neurologic outcome and controlled hyperthyroidism. This case not only suggests a new method for cooling treatment of thyroid storm but also provides evidence for TTM in patients who were resuscitated from IHCA and remained comatose after ROSC. However, Further wider studies are needed to confirm the results achieved and to obtain a standardization of the treatment.

## Data Availability Statement

The original contributions presented in the study are included in the article/Supplementary Material, further inquiries can be directed to the corresponding author/s.

## Ethics Statement

Written informed consent was obtained from the individual(s) for the publication of any potentially identifiable images or data included in this article.

## Author Contributions

YF wrote the initial draft for this case and this was corrected and reviewed by HG and QM. YZ, YL, BM, and WS participated in treatment and provided information about the patient. SL provided part of references. All authors have read and approved the manuscript.

## Conflict of Interest

The authors declare that the research was conducted in the absence of any commercial or financial relationships that could be construed as a potential conflict of interest.

## Abbreviations

AF: Atrial fibrillation
VT: Ventricular tachycardia
VF: Ventricular fibrillation
TdP: Torsade de pointes
FT3: Free triiodothyronine
FT4: Free thyroxine
TSH: Thyroid-stimulating hormone
TRAB: TSH-receptor antibody
ECG: Electrocardiogram
CPR: Cardiopulmonary resuscitation
ROSC: Return of spontaneous circulation
GCS: Glasgow coma scale
EICU: Emergency intensive care unit
TGAb: Thyroglobulin antibodies
TMAb: Thyroid microsomal antibody
TTM: Targeted temperature management
OHCA: Out-of-hospital cardiac arrest
IHCA: In-hospital cardiac arrest
JTA/JES: Japan Thyroid Association and the Japan Endocrine Society
CPC: Cerebral performance category
ACC/AHA/HRS: American College of Cardiology/American Heart Association/Heart Rhythm Society
ICD: Implantable cardioverter defibrillator.
